# ﻿Rediscovery of a second species of Reed Beetle in Israel (Coleoptera, Chrysomelidae, Donaciinae)

**DOI:** 10.3897/zookeys.1252.149428

**Published:** 2025-09-19

**Authors:** David G. Furth, Ariel-Leib-Leonid Friedman, Tamar Feldstein-Farkash

**Affiliations:** 1 Department of Entomology, Smithsonian Institution, Washington, D.C., USA; 2 Steinhardt Museum of Natural History, Tel Aviv University, Tel Aviv, Israel; 3 School of Zoology, Tel Aviv University, Tel Aviv, Israel

**Keywords:** Conservation, Donaciinae, Dora Park, indoor collecting, land reclamation/restoration, molecular taxonomy, wetland drainage

## Abstract

A species of reed beetle, *Donacia simplex* (Coleoptera, Chrysomelidae, Donaciinae) was recently rediscovered in northern Israel. Now a second species, *Donacia tomentosa*, has been rediscovered (verified using molecular analysis) living further south in Israel. In 2021 a reed beetle expert discovered a very old record of *Donacia tomentosa* from modern day Israel in the collections of the Natural History Museum (London) from a few specimens collected near Tel Aviv by the British in 1918 during WWI. In 2022 the authors discovered a viable population of *D. tomentosa* in Israel. Given the extensive amount of collecting by many good field entomologists in that region since 1918, it is surprising that this species had not been seen for 104 years. The location, biology, molecular analysis, and other details of this newly found viable population of *D. tomentosa* in Israel are provided here.

## ﻿Introduction

**Figure 1.**
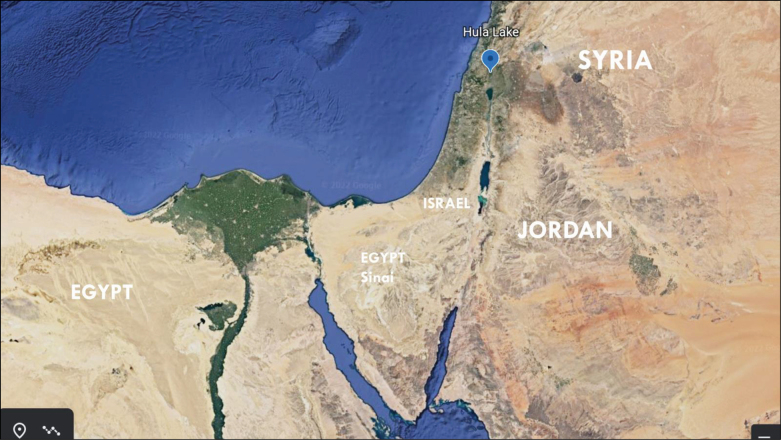
Google Earth Map of Middle East with Hula Valley site of *D. simplex* (Furth et al. 2023).

Prior to the senior author’s previous publications (Furth 1977, 1993), only *Donacia bicolora* Zschach (Coleoptera, Chrysomelidae, Donaciinae) had been recorded from modern day Israel and based on these publications it was recorded in Löbl and Smetana (2010). However, Furth et al. (2023) documented the discovery of *Donacia simplex* (Fabricius) in the Hula Valley, northern Israel and provided new observations and used molecular analysis to verify the identification (Fig. 1). *Donacia bicolora* and its food plant were thought to have been extirpated, along with some other animals and plants, because of the drainage of the Hula wetlands (lake and surrounding swamps) in northern Israel between 1951 and 1958 (Furth 1977). *Galerucella nymphaeae* Linnaeus (Chrysomelidae, Galerucinae) was also similarly apparently extirpated from the Hula Valley (Furth 1993). Furth (1977) mentioned a record of *D. thalassina* Germar from the Hula, but the specimen was later (Furth 1993) corrected by an expert (I. Askevold) as *D. marginata* Hoppe. Both *D. bicolora* and *D. marginata* (not collected from Israel since the 1940s) are known to feed on the same species, i.e., *Sparganium erectum* Linnaeus (Typhaceae) or congeneric food plants.

As mentioned in Furth et al. (2023), there has been some confusion regarding the image (fig. 3 of *Donacia bicolora* Zschach) in Furth (1977). Based on the subject of that publication, some readers may have understood that the specimen shown in Furth’s (1977) fig. 3 was from Israel. In the third paragraph of the discussion, Furth et al. (2023) mentioned that specimen was not from Israel and that it was only selected as an example of *D. bicolora* from the collections in the Museum of Comparative Zoology, Harvard University (MCZ). To completely clarify this oversight in the geographical designation of this specimen caused by the earlier publication (Furth 1977), the first author (DF) went to the MCZ in October 2023 and found this original specimen (Figs 4, 5).

This study and the previous ones demonstrate the importance of what the senior author (DF) has referred to as “indoor collecting” (Furth 2013); that is, the discovery of historical specimens in museum collections that often shed important light (e.g., new distribution records such as presence/absence of species in more modern times or even new species to science). In the current example Elisabeth Geiser discovered historical specimens in a collection of a species not previously recorded from Israel (Geiser and Jäch 2021). Such discoveries will certainly increase as collections are digitized, as well as with increasing “indoor collecting”. Such discoveries are of increasing importance as it becomes more difficult to perform fieldwork due to more stringent rules with regard to obtaining collecting and export permits, as well as new laws about access and benefit sharing.

*Donacia tomentosa* had not been recorded in Israel during recent history despite many excellent local and foreign entomological collectors who worked in modern day Israel. The specimens discovered in the historical collections of the Natural History Museum (London) by E. Geiser are the first records of this species and their data as reported in Geiser and Jäch (2021); the label data of these records are: “Palestine, nr. [near] Jerisheh, ca. 4–7 m, NE of Jaffa, 26.IV.1918, [det.? coll.?] S.L. Partbury 1969, det. E. Geiser 2019” (BMNH) (4 specimens). Jarisha (also Jerisha or Jerisheh) was a village located near the ancient site of Tell Jarisha (Tel Gerisa) on the south bank of Al-Awja (= Yarkon) River. After consultation (E. Geiser and I. Askevold pers. comms.) the “ca. 4–7 m” in this label data could be interpreted as elevation; however, it is more likely to refer to the distance of this site from Jaffa and therefore, should be “ca. 4–7 km”.

**Figure 2.**
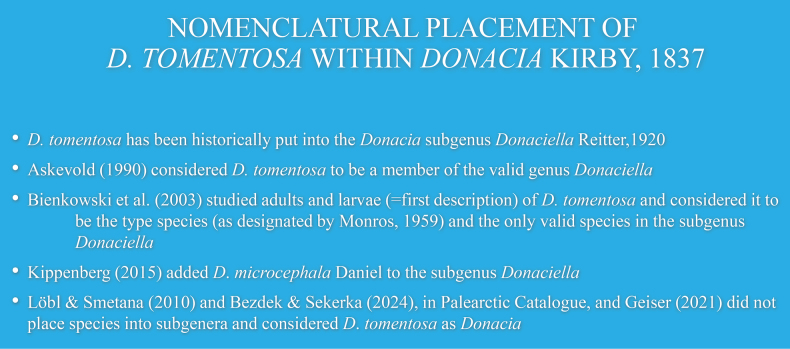
*Donacia tomentosa* nomenclature.

Bieńkowski et al. (2003) examined both adults and larvae of several species of *Donacia* and concluded that *D. tomentosa* should be considered the type species of a valid genus *Donaciella* Reitter, which had formerly been treated as a subgenus of *Donacia*. Bieńkowski et al. described the larvae of *D. tomentosa*. Kippenberg (2015) also placed *Donacia microcephala* Daniel in *Donaciella*, which he considered to be a valid subgenus (not a genus), meaning that there were two species in *Donaciella*. Even though Askevold (1990) considered *Donaciella* to be a valid genus, most workers still consider it to be a subgenus of *Donacia* (Fig. 2).

**Figure 3.**
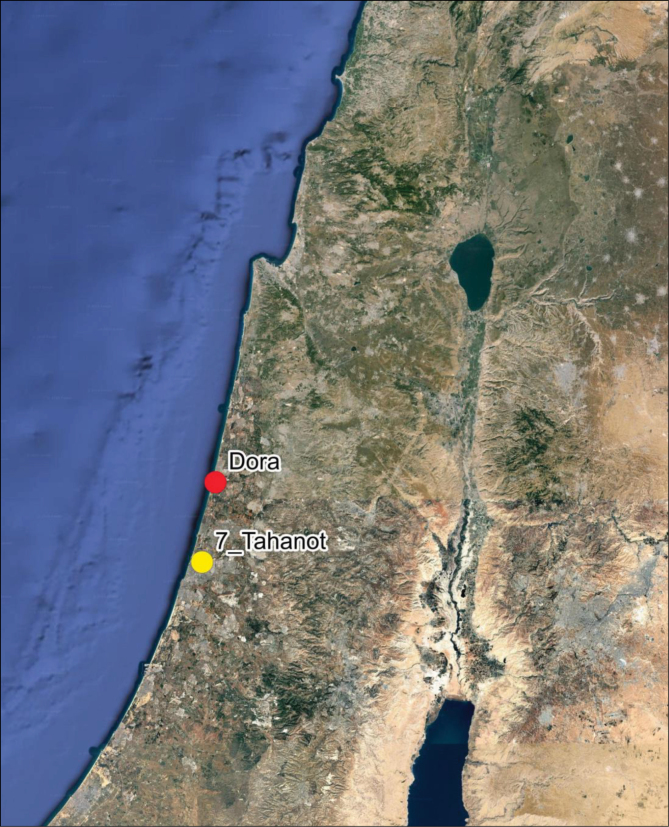
Google Earth Map showing the original 1918 location of *D. tomentosa* (7-Tahanot) and its current location (Dora Park) in Israel (prepared by Z. Yanai).

**Figure 4.**
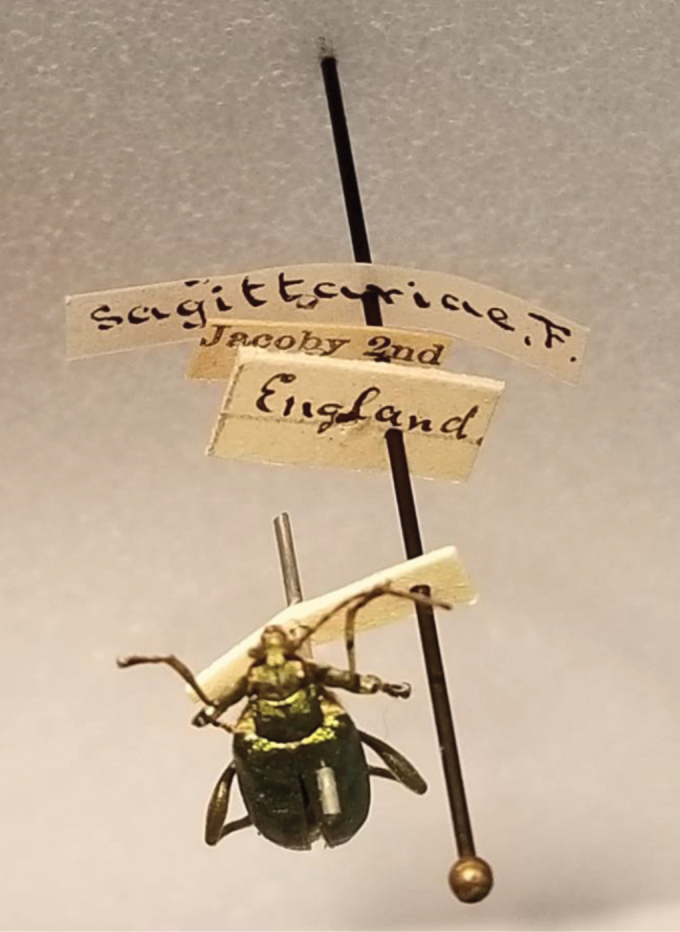
*Donacia bicolora* specimen at Museum of Comparative Zoology of Harvard University, in Furth (1977: fig. 3) (photo: D. Furth).

**Figure 5.**
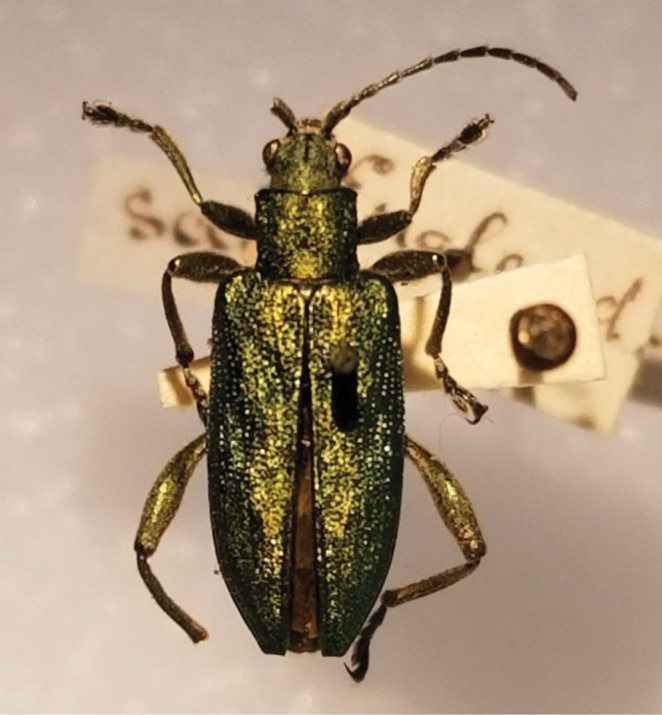
*Donacia bicolora* specimen labels at Museum of Comparative Zoology of Harvard University, in Furth (1977: fig. 3) (photo: D. Furth). [Museum of Comparative Zoology, Harvard University@President and Fellows of Harvard College. https://mcz.harvard.edu/permissions-copyright]

The following list included *Donacia* species recorded nearest to Israel (Bezděk and Sekerka 2024), updated from Geiser and Jäch (2021)* and Geiser (2024b)**. As mentioned above, *D. bicolora* was also recorded from Israel by Furth (1977, 1993):

- *D. bicolora*: Iran; Lebanon (E. Geiser pers. comm.); Israel; Turkey; Albania*; Greece**; Serbia*; Bosnia-Herzegovina*; Montenegro*.
- *D. marginata*: Iran; Israel; Greece: Turkey; Morocco; Bosnia-Herzegovina*; Serbia*.
- *D. simplex*: Turkey; Algeria; Morocco; Croatia; Bosnia-Hercegovina**; Albania**; Bulgaria; Serbia*; Greece**; Iran*; Syria (Anti-Lebanon mountains)*; Lebanon (E. Geiser pers. comm.).
- *D. tomentosa: Donacia tomentosa* distribution according to Löbl and Smetana (2010): Azerbaijan; Belgium; Bosnia-Herzegovina; Bulgaria; Belarus; Russia (Central European Territory); Czech Republic; Estonia; Finland; France; Germany; Hungary; Italy; Latvia; Lithuania; Luxembourg; Netherlands; Poland; Romania; Russia (North, Central and Southern European Territories and West Siberia); Slovakia; Slovenia; Sweden; Switzerland; Ukraine; Kazakhstan; Turkmenistan; Turkey; Uzbekistan. Geiser and Jäch (2021) added new records from Austria; Crimea; Croatia; Greece; Iran; Israel. Note: the distribution of *D. tomentosa* in Iran is actually at locations in northwestern Iran (e.g., Guilan and Lorestan Provinces); therefore, this species is probably not throughout the entire country (Geiser and Jäch 2021), although the faunistic knowledge of Iran is poor for Chrysomelidae and *D. tomentosa* is not widespread even in regions where it occurs (E. Geiser pers. comm.).

Askevold (2024) alluded to a similarly rare, possibly extirpated, species in North America (*Plateumaris schaefferi* Askevold) that was recently rediscovered on the iNaturalist platform based on three separate observations. Askevold postulated that beavers may have played a role in re-creating habitat as they recovered from near-extirpation themselves. In iNaturalist there is a vast repository of data pertaining to the existence and distribution of species, as well as natural history information which does not usually accompany dead museum specimens. Currently, there are 10,795 observations by over 4,800 observers documenting 94 species of Donaciinae from around the world (I. Askevold pers. comm.).

Typical host plant damage for many species of *Donacia* is not very well documented in the literature, and some experts have not paid much attention to this subject (I. Askevold pers. comm.). However, Furth et al. (2023) the presumed host plant damage of *D. simplex* (Fabricius) on *Sparganium erectum* was shown in Fig. 7. For examples of *Donacia* species food plant damage, and at the suggestion of I. Askevold (pers. comm.), DF searched through an iNaturalist link that only illustrated some general photos identified as *Donacia* spp. After examining over 200 photographs by observers mainly in Europe and North America on iNaturalist DF found quite a few images of various *Donacia* species (e.g., *D. marginata* Hoppe, *D. vulgaris* Zschach, *D. bicolora*, etc.) of hostplant damage that matched the kinds of damage reported by Furth et al. (2023), as well as the damage observed in the current study of *D. tomentosa*.

Furth et al. (2023) also discussed the idea to use recent study of *D. simplex* to try to determine locations where that newly rediscovered species could be re-introduced into places that are protected, such as nature reserves or national parks. The current study sheds even more light on this idea.

## ﻿Materials and methods

See also details in the Introduction section above.

The bulk of the specimens of *Donacia tomentosa* in this study were collected by sweep netting at Dora Park (32°17'29"N, 34°50'49"E; Netanya, Israel) on April 25, 2022, and April 25, 2024. However, the second author (LF) collected the first specimens on April 18, 2022, as well as the dates listed below, and DF accompanied him on two occasions. The specimens were either placed directly into 95% ethanol or put into 95% ethanol shortly after collecting. Some specimens were also pinned and deposited in the collections at the Steinhardt Museum of Natural History of Tel Aviv University (**SMNH TAU**) and the U. S. National Museum of Natural History, Smithsonian, Washington DC (**USNM**). Several live specimens were given to A. Weinstein for high-resolution photos. Some specimens from April 25, 2022 were subjected to molecular analysis.

Fieldwork that found *D. tomentosa* at Dora Park (Netanya, Israel; 32°17'29"N, 34°50'49"E):

- April 18, 2022, LF collected the first specimens
- April 25, 2022, DF and LF collected
- April 5, 2024, LF collected
- April 17, 2024, LF collected
- April 25, 2024, LF and DF collected
- May 5, 2024, LF collected

Fieldwork by DF and LF searching the original recorded locality (Geiser and Jäch 2021) at “Tel Jarisha”, 1918 locality (32°05'47"N, 34°48'29"E):

- April 24, 2023 – LF and DF visited Sheva’ Tahanot (7 Stations or “Tel Jarisha”) along the Yarkon River (Tel Aviv) to document presence of *Butomus* and/or *D. tomentosa* (Fig. 3).

Fieldwork to Berekhat Ya’ar Nature Reserve (32°24'39"N, 34°53'52"E):

- May 4, 2023, DF and LF visited Berekhat Ya’ar to document the presence of *Butomus* and *Sparganium* and to check for the presence of *Donacia* species (*D. tomentosa* or *D. simplex*).

Museum visit: October 3, 2023, DF visited the Museum of Comparative Zoology of Harvard University (**MCZ**) to find specimen of *D. bicolora* (from Furth 1977: fig. 3, with no geographic information) and to document its locality.

Barcoding *Donacia* samples DNA was extracted from a hind leg of four individuals voucher numbers SMNHTAU: In.426060-3 collected by Dr David Furth. Samples were digested overnight at 60 °C, and the extraction continued following the manufacturers’ protocol (Genomic DNA Mini Kit (Tissue); GT100, Geneaid). A fragment of 658 bp of the mitochondrial cytochrome c oxidase I (COI) was amplified and sequenced with the primers LCO_1490 and HCO_2198 (Folmer et al. 1994). PCR conditions consisted of 2 min initial denaturation at 95 °C followed by five touchdown cycles: 15 s denaturation at 95 °C, 15 s annealing at 55 °C down to 51 °C (Δt = −1 °C each cycle), 30 s extension at 72 °C, followed by additional 35 cycles of 15 s denaturation at 95 °C, 15 s annealing at 50 °C, 30 s extension at 72 °C, and final extension at 72 °C for 5 min (using PCRBIO HS Taq Mix Red; PCRBIOSYSTEMS). PCR products were verified on a 1% TAE agarose gel and amplifications were sequenced at Microsynth AG (Germany) in both directions using the PCR primers. Sequences were deposited in GenBank under accession numbers PQ878470–PQ878473.

The sequences were submitted to a blastn search (on April 18, 2023; BLASTN 2.14.0+ Zhang et al. 2000). The blastn results showed that the sequences were 97% identical either to *D. simplex* (for vouchers SMNHTAU: In.426062-3), or to *D. tomentosa* (for vouchers SMNHTAU: In.426060-1). All sequences of *Donacia* available in GenBank were retrieved (April 2023; see Suppl. material 1), as well as outgroup sequences of *Plateumaris braccata* (following Furth et al. 2023). Sequences of *Donacia hirticollis* were discarded from the analysis because they were distant from all other *Donacia* sequences. The sequences were aligned by MAFFT 7.304 (Katoh and Standley 2013) using LINS-i parameters. Phylogenetic tree (Fig. 17) was reconstructed based on COI sequences (568 bp) under the maximum-likelihood criterion with PhyML 3.0 (Dereeper et al. 2008; Guindon et al. 2010) using the GTR model of sequence evolution. Bootstrap percentages computed based on 100 replicates. The sequences of specimens SMNHTAU: In.426060-1 cluster well within *D. tomentosa* from Europe, while SMNHTAU: In.426062-3 cluster with *D. simplex* with a bootstrap support of 98%.

For examples of *Donacia* species food plant damage, and at the suggestion of I. Askevold (pers. comm.), DF searched through an iNaturalist link that only illustrated some general photos identified as *Donacia* spp.

## ﻿Results

During an October 3, 2023 visit to the MCZ, DF was able to locate and photograph the original *D. bicolora* specimen pictured in his previous publication (Furth 1977: fig. 3); it proved to be from England, not Israel (Figs 4, 5). Its original labels are “England; Jacoby 2^nd^ Collection; sagittariae F.”.

**Figure 6.**
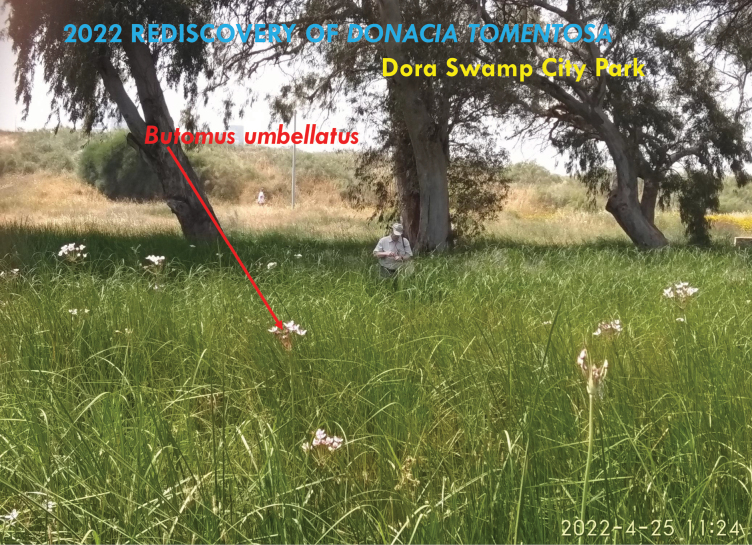
D. Furth collecting at Dora Park, April 25, 2022 (photo: L. Freidman).

**Figure 7.**
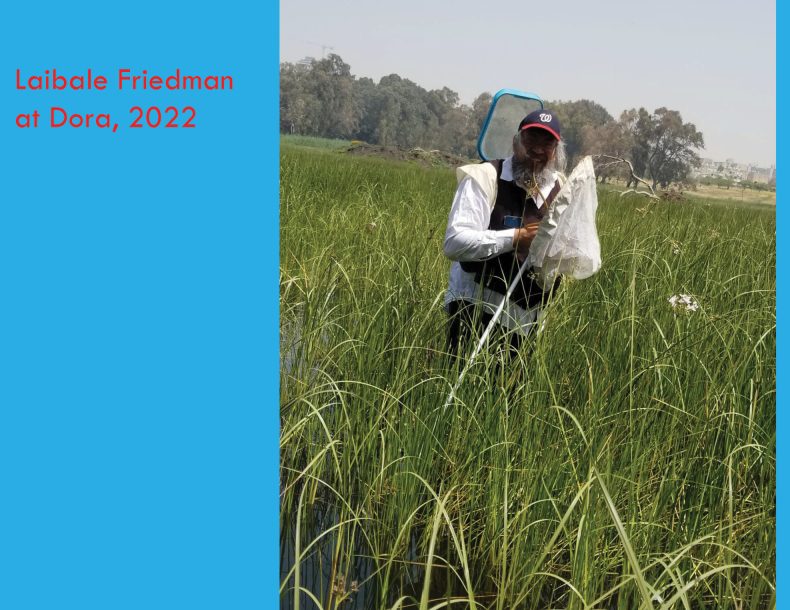
L. Friedman collecting at Dora Park, 25 April 2022 (photo: D. Furth).

**Figure 8.**
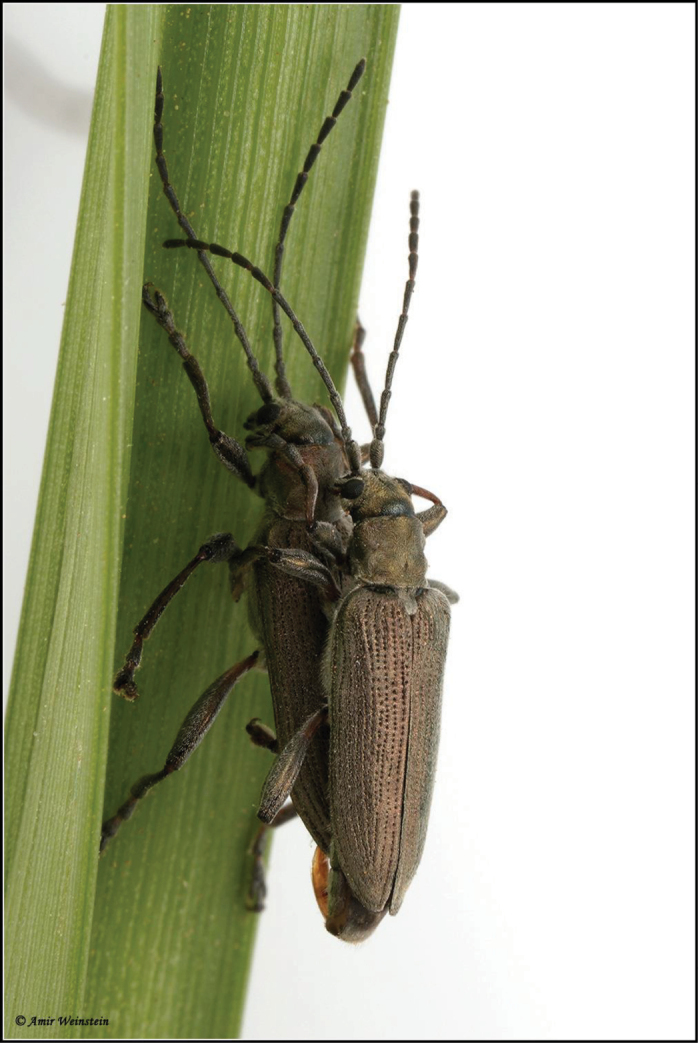
*Donacia tomentosa* adults live, in copula (photo: A. Weinstein).

**Figure 9.**
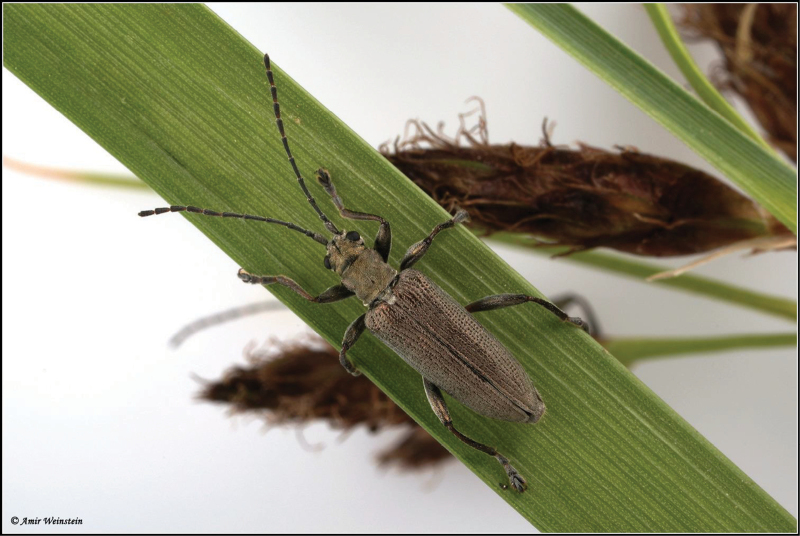
*Donacia tomentosa* adult live (photo: A. Weinstein).

**Figure 10.**
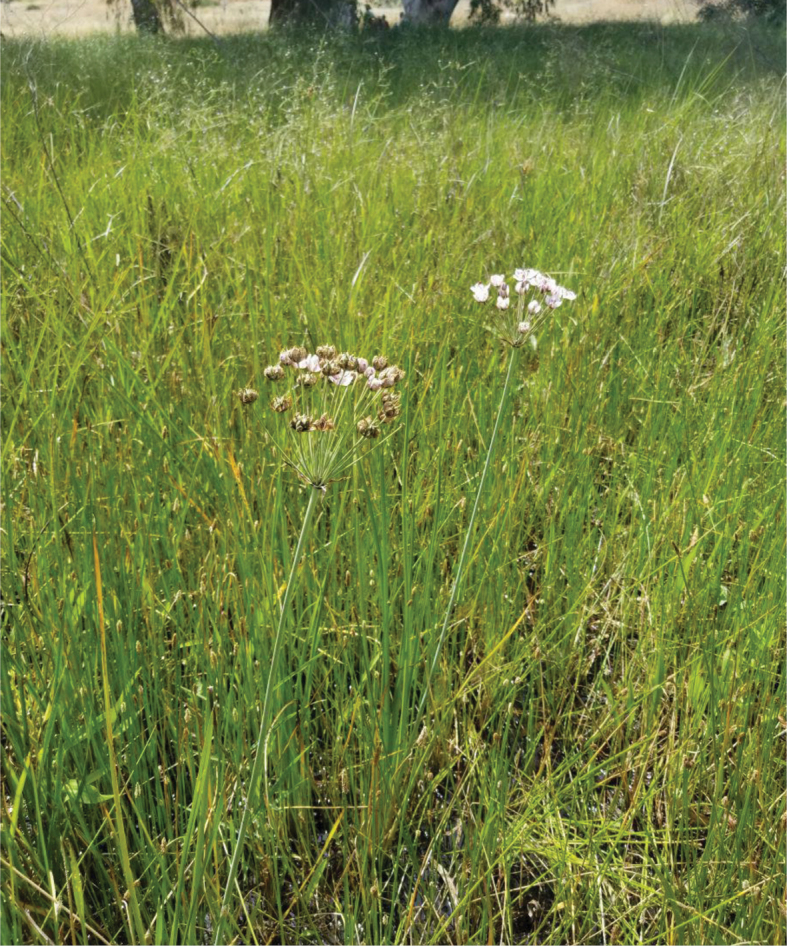
*Butomus umbellatus* at Dora Park (photo: D. Furth).

**Figure 11.**
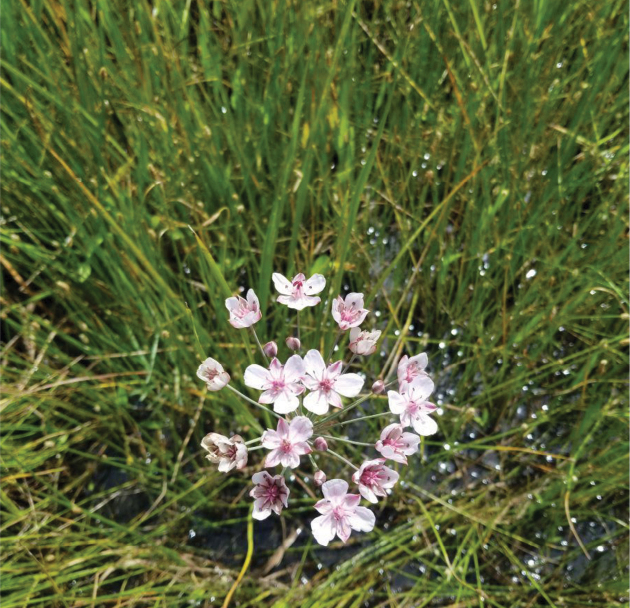
*Butomus umbellatus* flower at Dora Park (photo: D. Furth).

**Figure 12.**
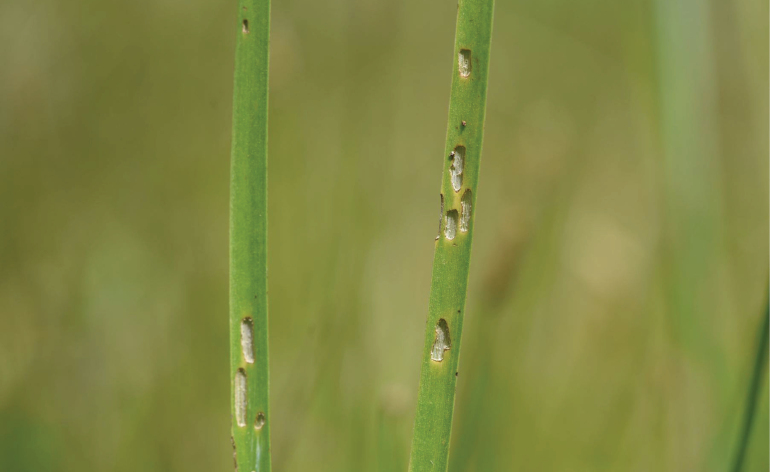
*Donacia tomentosa* adult feeding on leaf of *Butomus umbellatus* (photo: O. Auster).

The current project began when the second author (LF) was searching for a species of semi-aquatic weevil (Bagous (Macropelmus) validus Rosenhauer; Coleoptera, Curculionidae) that feeds on *Butomus umbellatus* L. (Butomaceae) and until recently was found occurring in Israel only at the Berekhat Ya’ar swamp (Friedman 2018). On April 18, 2022 at Dora Park in the city of Netanya, LF collected two specimens that he thought might be *Donacia simplex* which had been reported from northern Israel by Furth et al. (2023). LF was very familiar with the previous study because he helped DF search for and collect *D. simplex* on multiple occasions. LF called DF from the field (still standing in the water) thinking that it was *D. simplex*. The DF was surprised, since neither *D. simplex* nor its host plant, *Sparganium erectum*, had ever been recorded near Dora Park. Therefore, a few days later (April 25) LF and DF went to Dora Park to try to collect more specimens. On April 25 at Dora Park both LF and DF collected multiple specimens and kept some alive for observation and photography. At that time these authors suspected that this *Donacia* was feeding on *Bolboschoenus maritimus* (L.) Palla (Cyperaceae) (formerly in the genus *Scirpus*), because they collected them by sweeping this plant that was a dominant plant in this Dora Park wetland (Figs 6, 7). A significant number of the specimens were in copula. The same day (April 25) a few live specimens were given to a colleague (an excellent photographer, Amir Weinstein) who took them home to photograph (Figs 8, 9). Subsequently the photos were sent to Elisabeth Geiser (Vienna, Austria), an expert on Donaciinae . From the clear photographs E. Geiser immediately recognized this species as *Donacia tomentosa* Ahrens (E. Geiser pers. comm) and said that it was a rare species in many parts of Europe and that its recorded host plant is *Butomus umbellatus*. She also told us that she had just published a record of this species from Israel based on specimens she had recently found in the Natural History Museum (London) (Geiser and Jäch 2021). Based on this information DF and LF returned to Dora Park the next year (2023) to collect more specimens by sweeping *B. umbellatus* (Figs 10, 11). No specimens of this species exist in the Israel National Insect Collection at SMNH TAU. The feeding damage on leaves of the host plant *B. umbellatus* was also documented (Fig. 12).

**Figure 13.**
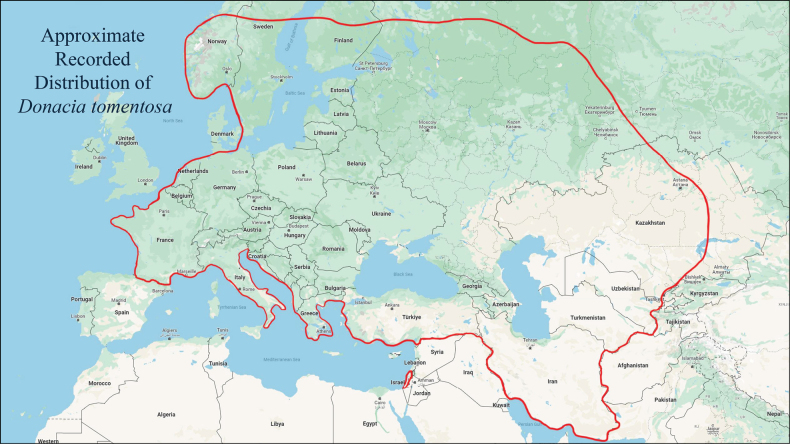
Distribution of *Donacia tomentosa* (Löbl and Smetana 2010; Geiser and Jäch 2021) (Map by K. Darrow).

*Donacia tomentosa* has a relatively wide distribution in western and parts of eastern Europe (Fig. 13). Its presence in Israel is considerably disjunct and south of its previously known recorded distribution (Geiser and Jäch 2021; Geiser 2024a). The two new records reported by Geiser and Jäch (2021) from Iran are Fuman, Gilan Province (37°7'48"N, 49°11'24"E) on the southwestern coast of the Caspian Sea in northwestern Iran (ca 3340 km northwest of Tehran) and Borujerd, Lorestan Province (33°30'N, 48°30'E) also in northwestern Iran, 520 km south of Fuman. These Iranian localities of *D. tomentosa* are north of the latitude of the Israeli localities of the species.

During the collecting visit to the 1918 site “Tel Jarisha” (now Sheva’ Tahanot in Tel Aviv) on April 24, 2023, DF and LF did not find any *Donacia* spp., and they did not observe any *Butomus umbellatus*.

**Figure 14.**
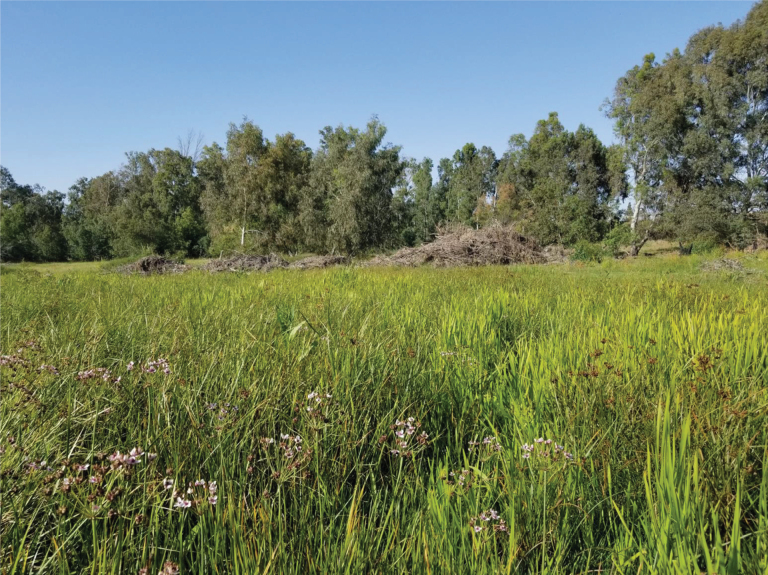
*Butomus umbellatus* at Berekhat Ya’ar Nature Reserve (photo: D. Furth).

**Figure 15.**
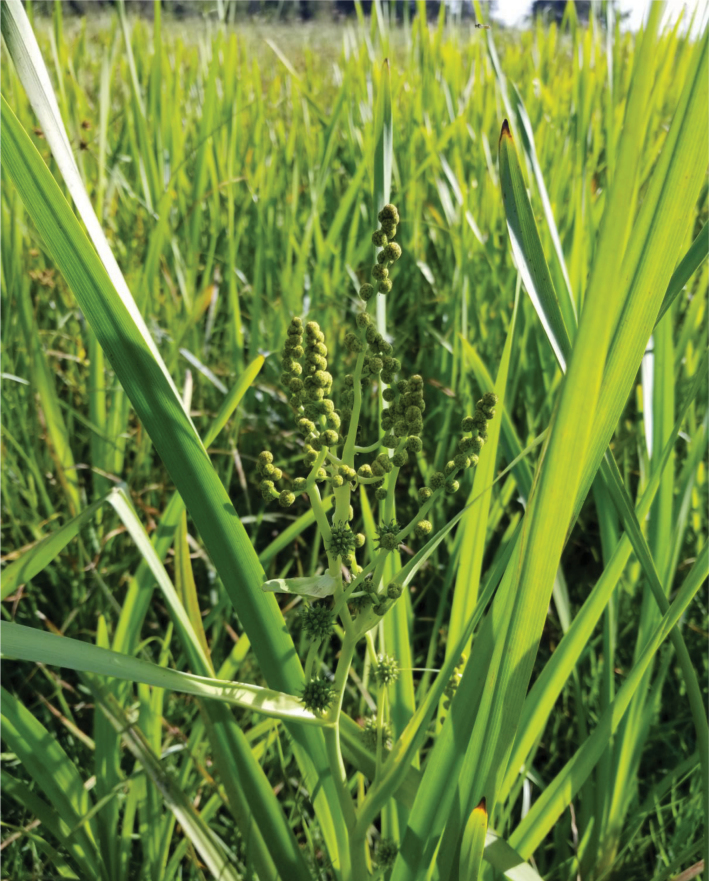
*Sparganium erectum* fruits at Berekhat Ya’ar Nature Reserve (photo: D. Furth).

Zohar Yanai (pers. comm. 2023) documented *Sparganium* at Berekhat Ya’ar in boggy areas with only small areas of open water. During a May 4, 2023 visit to Berekhat Ya’ar by DF and LF no specimens of *Donacia* spp. were found, although both *B. umbellatus* and *S. erectum* were observed (Figs 14, 15). The Israel Ministry of the Interior very recently officially declared Berekhat Ya’ar to be a nature reserve under the protection of the Israel Nature and Parks Authority (Yariv Malihi and Zohar Yanai pers. comms.).

As observed by Furth et al. (2023) for *D. simplex*, sequences of *D. tomentosa* from Israel exhibit greater divergence in France and Finland. This nucleotide distance is likely influenced by the geographic separation.

## ﻿Discussion

The rediscovery of *Donacia tomentosa* in present-day Israel after 104 years is interesting and revealing. There have been many excellent entomological collectors, both local and foreign, in Israel and surrounding areas since 1918. Both DF and LF have extensively collected there over the past 52 and 35 years, respectively. Additionally, older local entomologists such as F. Bodenheimer, O. Theodor, H. Bytinski-Salz, J. Kugler, J. Wahrman, M. Pener, L Fishelson, Y. Palmoni, J. Halperin, A. Freidberg, and A. Gasith, among others, since the 1920s. They and many other local and foreign entomologists have collected in wetland habitats similar to the Dora Park without discovering *D. tomentosa*!

The results of this study have revealed that even after the complete alteration of the habitat of its originally locality into a public park and its apparent 104-year absence in Israel, *D. tomentosa* persisted and its rediscovery means that at least two species of *Donacia* have recently been rediscovered in Israel despite the anthropogenic changes over the past century. Other *Donacia* species may still exist in Israel (e.g., *D. bicolora* and *D. marginata*). Similar to *D. simplex* rediscovered in northern Israel at Kibbutz Dan (Furth et al. 2023) the long term preservation of *D. tomentosa* is in danger since its only known population is not in a protected area. There need to be attempts to introduce both of these *Donacia* species to preserves that are permanently protected along with their food plants. Therefore, the current study and the previous one (Furth et al. 2023) demonstrate the resilience of the fauna and flora despite significant habitat alteration by humans. Nevertheless, it is important to try to preserve such rare biota and even to re-introduce species into protected areas like nature reserves. The discovery of the *Sparganium erectum* and *Butomus umbellatus*, the food plants of *D. simplex* and *D. tomentosa*, respectively, at Berekhat Ya’ar Nature Reserve is encouraging for the potential re-introduction and establishment of these two rediscovered *Donacia* species in Israel. Dr E. Geiser (pers. comm.) mentioned that *B. umbellatus* prefers such ecological conditions and *D. tomentosa* needs this as a food plant. In fact, *B. umbellatus* tolerates changing water levels much more than other plants and is, therefore, more competitive in such locations. Although *B. umbellatus* and *D. tomentosa* can be found in wetlands that seasonally dry up, it is not certain that such habitat is suitable for *D. simplex* (E. Geiser pers. comm.). Because *Sparganium* spp. are not as rare as *B. umbellatus*, *D. simplex* is not as rare as *D. tomentosa*, as evidenced in museum collections (E. Geiser pers. comm.). Additional study of the life cycles and ecology of these two *Donacia* species, particularly *D. simplex*, is necessary and cooperation with the Israel Nature and Parks Authority is needed.

The records in Israel of two recently rediscovered *Donacia* species represent the southern geographic limits of these Palearctic species. Molecular analysis shows that these species’ populations in Israel are genetically distinct from populations in Europe, which reinforces their importance of their conservation.

Furth et al. (2023) previously demonstrated that at least *D. simplex* is still currently found in northern Israel despite the drainage of the Hula Lake and Swamps. With the current study we now know that a second species is currently still in Israel. Hopefully in the future we will be able to rediscover that *D. bicolora* has also survived the various anthropogenic changes in northern Israel. Previous identifications of *Donacia*, especially within the collections in Israel, were not actually determined by *Donacia* experts. The rediscovery of *D. tomentosa* demonstrates the value of combining research on natural history, ecological, collections-based study of specimens with molecular identification.

Even though Donaciinae species are primarily Holarctic, it is probable that other, more cryptic *Donacia* species, such as *D. marginata* or *D. bicolora*, may be discovered or rediscovered in the biogeographically diverse biotopes of Israel.

**Figure 16.**
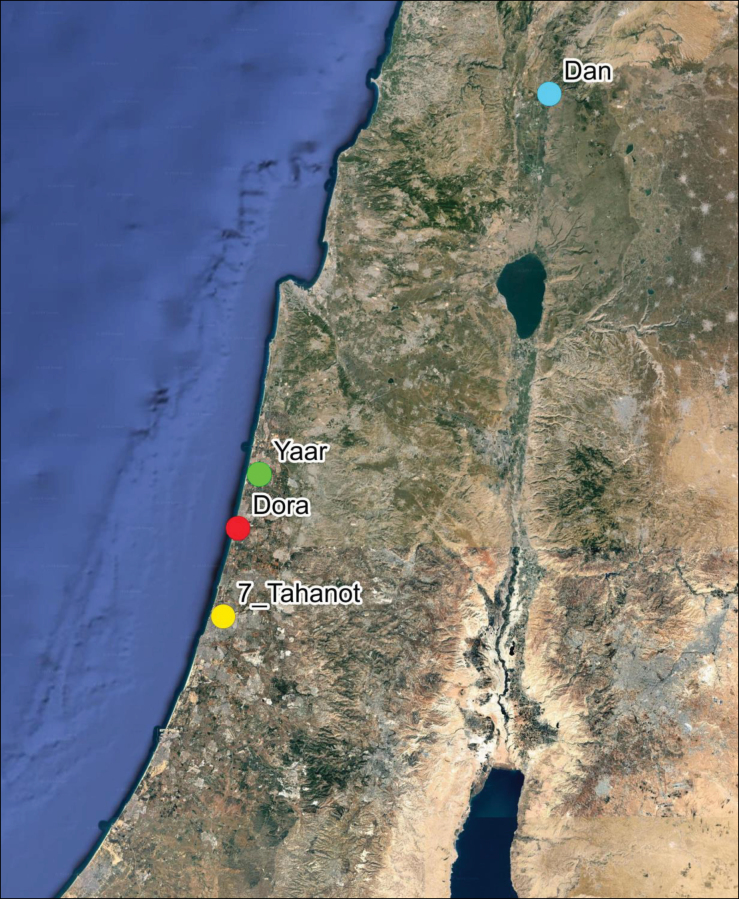
Map of the original 1918 site (Sheva’ Tahanot), the new site (Dora), and the potential re-introduction site (Berekhat Ya’ar); Hula (=Dan site of *D. bicolora*) (prepared by Z. Yanai).

As for *D. simplex* (Furth et al. 2023), the current study reveals the necessity for preserving populations of *D. tomentosa* as well as its food plant, *Butomus umbellatus*. Currently neither *D. simplex* nor *D. tomentosa* are in the list of the Endangered and Threatened species of Invertebrates of Israel, but an effort should be made to include them. Fortunately, the food plant of *D. simplex*, *S. erectum*, is listed as Vulnerable on the Red List of Endangered Plants of Israel and *Butomus umbellatus* is Endangered (Shmida and Pollack 2007; Yariv Malihi pers. comm.). As with *D. simplex*, a future goal should be to re-introduce *D. tomentosa* to locations where it can be protected. One potential re-introduction location is the Berekhat Ya’ar Nature Reserve (Fig. 16) where there is already a population of *B. umbellatus*. Therefore, the optimum re-introduction locations for *D. tomentosa* and *D. simplex* would be in protected areas.

**Figure 17.**
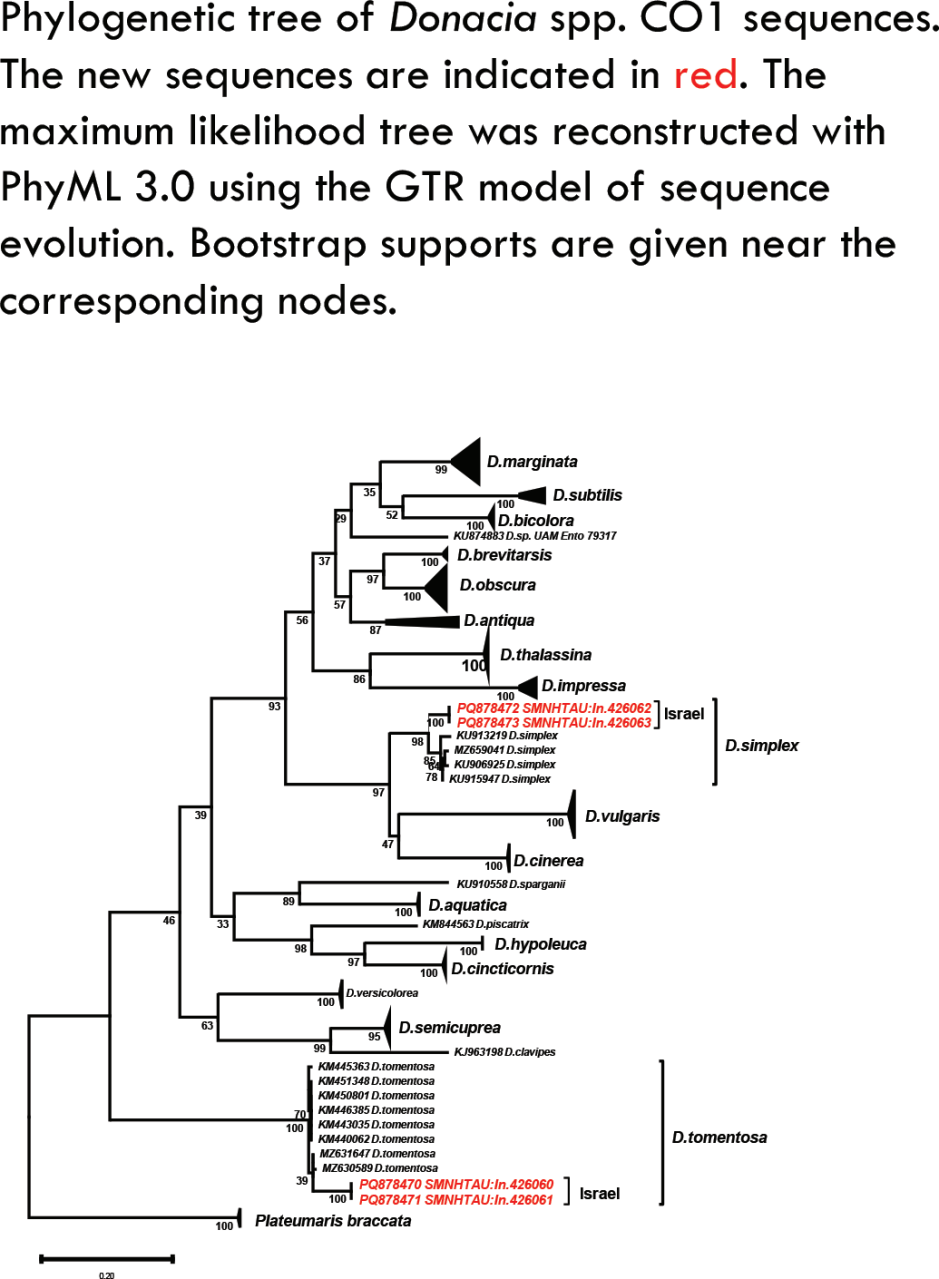
Phylogenetic tree reconstruction of the genus *Donacia* inferred using 658 bp of the COI of the mitochondrial gene. Terminal nodes are visually collapsed to the species level, except for *D. simplex*. The sequences of *Donacia* collected in Israel are reported in red. The tree scale bar indicates the distance in substitutions per site. Bootstrap support values (100 replicates) are shown below the nodes (analysis and tree by T. Feldstein-Farkash).

Based on studies of the biological and environmental conditions of water beetles of winter pools that were under threat by the construction of a light-rail system near Haifa, Israel, Avital Gasith (pers. comm. 2025) demonstrated the importance of preserving temporary winter pools. This study also illustrated the value of working together with the Israel Nature and Parks Authority and the Cross-Israel Highway Company to conduct ecological surveys as indicators to ensure the future preservation of these unique temporary pools. Similar studies regarding the introduction of *Donacia* species to Berekhat Ya’ar temporary wetland are probably warranted.

## ﻿Conclusion

The rediscovery of a second species of *Donacia* spp. in Israel is both exciting and encouraging. A species thought to have been extirpated by human activities and development has managed to survive. Of course, there are examples of such rediscoveries even with vertebrates. These instances also provide opportunities for better preservation of species that may be threatened or endangered, especially when herbivorous species are involved, and their host plant reduction may be the primary reason for threats to their existing in often small populations.

This specific case illustrates well the value of historical natural history collections to document the presence or absence of species at different points in time and that this kind of “indoor collecting” is as valuable and informative as the traditional outdoor collecting. *Donacia tomentosa* was discovered in an unprotected city park on its host plant (*Butomus umbellatus*) known from its more northern populations in Europe. It had not been collected and recorded in Israel or the nearby surrounding areas for 104 years yet somehow the species survived all the human disturbances of its habitats. The molecular analysis provided in this study documents the identity of *D. tomentosa*. The presence of the foodplant *Butomus umbellatus* as well as the foodplant (*Sparganium erectum*) of the recently rediscovered *D. simplex* in a nearby national park offers a potential habitat to introduce and preserve both species in a more protected location such as the Berekhat Ya’ar Nature Reserve.

## ﻿Additional information

### Author ORCIDs

David G. Furth https://orcid.org/0000-0001-6559-1209

Tamar Feldstein-Farkash https://orcid.org/0009-0007-9277-1417

### Data availability

All of the data that support the findings of this study are available in the main text or Supplementary Information.

## Supplementary materials

Supplementary material 1 All available sequences of Donacia retrieved in GenBank with their country location and GenBank accession numbers Data type xlsx https://binary.pensoft.net/file/1419963This dataset is made available under the Open Database License (http://opendatacommons.org/licenses/odbl/1.0/). The Open Database License (ODbL) is a license agreement intended to allow users to freely share, modify, and use this Dataset while maintaining this same freedom for others, provided that the original source and author(s) are credited.David G. Furth, Ariel-Leib-Leonid Friedman, Tamar Feldstein-Farkash
